# Sensorless Junction Temperature Estimation of Onboard SiC MOSFETs Using Dual-Gate-Bias-Triggered Third-Quadrant Characteristics

**DOI:** 10.3390/s25020571

**Published:** 2025-01-20

**Authors:** Yansong Lu, Yijun Ding, Jia Li, Hao Yin, Xinlian Li, Chong Zhu, Xi Zhang

**Affiliations:** School of Mechanical and Engineering, Shanghai Jiao Tong University, Shanghai 200240, China; yansong.lu@sjtu.edu.cn (Y.L.); dingyijun0715@sjtu.edu.cn (Y.D.); 18852647159@sjtu.edu.cn (J.L.); jsyinhao@sjtu.edu.cn (H.Y.); lixinlian@sjtu.edu.cn (X.L.); braver1980@sjtu.edu.cn (X.Z.)

**Keywords:** temperature monitoring, metal oxide semiconductor field-effect transistors (MOSFETs), silicon carbide (SiC), body diode, third-quadrant (3rd-quad) characteristics, electric vehicles (EVs)

## Abstract

Silicon carbide (SiC) metal oxide semiconductor field-effect transistors (MOSFETs) are a future trend in traction inverters in electric vehicles (EVs), and their thermal safety is crucial. Temperature-sensitive electrical parameters’ (TSEPs) indirect detection normally requires additional circuits, which can interfere with the system and increase costs, thereby limiting applications. Therefore, there is still a lack of cost-effective and sensorless thermal monitoring techniques. This paper proposes a high-efficiency datasheet-driven method for sensorless estimation utilizing the third-quadrant characteristics of MOSFETs. Without changing the existing hardware, the closure degree of MOS channels is controlled through a dual-gate bias (DGB) strategy to achieve reverse conduction in different patterns with body diodes. This method introduces a MOSFET operating current that TSEPs are equally sensitive to into the two-argument function, improving the complexity and accuracy. A two-stage current pulse is used to decouple the motor effect in various conduction modes, and the TSEP-combined temperature function is built dynamically by substituting the currents. Then, the junction temperature is estimated by the measured bus voltage and current. Its effectiveness was verified through spice model simulation and a test bench with a three-phase inverter. The average relative estimation error of the proposed method is below 7.2% in centigrade.

## 1. Introduction

There have been increasing demands on the thermal safety of electric vehicles (EVs) to make transportation more reliable and sustainable, especially since tremendous progress has been made in power semiconductor devices, achieving higher power ratings in more compact dimensions with lower total weight [1]. Silicon carbide (SiC) power metal oxide semiconductor field-effect transistors (MOSFETs) represent a new generation of wide-bandgap semiconductors due to their significant advantages over silicon insulated gate bipolar transistors (IGBTs) in terms of excellent switching characteristics, low losses, high operating temperatures, and high blocking voltages [2], and they are being increasingly applied in EVs [3].

Freewheeling through intrinsic diodes in power converters is a cost-effective solution without sacrificing conversion efficiency [4]. Regarding traction inverters, the reverse conduction loss can be suppressed by shortening the dead time to achieve a higher power factor [5]. The third-quadrant (3rd-quad) characteristic of MOSFETs represented by the body diode meets the freewheeling demand of the traction inverter during the dead time, and significant relevant research efforts have been devoted to this topic. The reverse conduction path of a MOSFET in the third quadrant includes a body diode with a PN junction, along with a parallel MOS channel [6]. The reverse conduction of MOS channels with a positive gate bias can reduce losses [7], while a zero or negative gate bias may not close the MOS channel completely, indicating that the third quadrant is not just about body diodes [8]. The MOS channel current is affected not only by the dynamic threshold voltage [9] but also by the gate bias and junction temperature [10]. The increase in junction temperature leads to a decrease in the threshold voltage, thus reducing channel resistance [11]. Still, the forward voltage drop of the body diode consisting of the PN junction is lower and proportional to temperature and thus tends to obtain more current [12]. Therefore, the current change is a complicated competition between MOS channels and intrinsic body diodes.

Multiple techniques have been developed to measure or estimate the junction temperature of power semiconductors. Firstly, direct measurement is an intuitive method, such as methods based on optical features [13] and thermistors connected with wafers [14]. However, they are limited by space, the packaging, and the cooling structure of the device. In addition, temperature-sensitive electrical parameters (TSEPs) as an indirect measurement method have different characterization indicators based on the temperature behaviors of semiconductors. The quasi-threshold voltage is captured at the moment of the voltage drop on parasitic inductance between the power source terminal and auxiliary source terminal by the designed circuit [15]. Measuring the gate internal resistance (i.e., peak current) requires real-time current peak detection and integrating circuits [16]. The switching delay time is extracted by an edge-detection-based measurement circuit with picosecond resolution [17], and the maximum current switching rate [18] is also based on a high-bandwidth device. The most common method is to detect the on-state features as indicators. The on-resistance is inferred based on the on-state voltage drop measured by a circuit with an auxiliary MOSFET [19]. The temperature sensitivity of the drain–source voltage and source–drain voltage was calibrated at low currents below 1 A, taking into account the influence of the gate–source voltage [20]. The influence of different SiC MOSFET process structures on source–drain voltages under conditions below 0.1 A was considered in [21]. Hu et al. [22] proposed a method based on a dedicated calibration circuit that decouples the switching and conduction losses to monitor the on-state voltage. In addition, the on-resistance was measured to detect its temperature and healthy state through a non-invasive monitoring circuit designed independently of information on the load and gate driver [23]. These TSEPs are all features of MOSFETs. Equally, there has been some TSEP-related research on diodes, such as studies measuring the turn-off reverse recovery current based on the high-voltage-withstand injection circuit and sampling circuit [24] and the turn-on delay time and forward voltage drop of the intrinsic body diodes of MOSFETs [25] by using a chip-integrated sensor [26]. However, these TSEP methods require an additional high-resolution measurement device, which would be integrated into the board, inevitably causing complicated implementation and reduced system reliability. In general, they focus on a single indicator of the device while neglecting that the estimated target of all indicators is consistent, i.e., the wafer temperature. Research on junction temperature estimation still lacks consideration of the combined TSEPs of MOSFETs and intrinsic body diodes.

For the temperature of a drive system, Dianov [27] developed an injection method to estimate the stator winding temperature, while Lu et al. [28] proposed the temperature co-estimation of an IGBT and stator winding considering the resistance ratio of the inverter and motor, but with a simplified current factor. The lumped parameter thermal network (LPTN) model based on power loss, whose parameters can be extracted from large-scale finite element method (FEM) simulations [29] or datasheet values, can be used to simplify the modeling process. Although the cost is lower, in practical applications, the thermal conductivity ratio between physical layers must be considered in an LPTN to optimize the heat flow path [30]. Although some models have considered thermal effects [31], previous studies have mainly focused on materials and thermodynamics, neglecting the electrical properties of SiC MOSFETs and their influence factors, and the features of actual gate drivers.

This paper proposes a datasheet-driven method for the sensorless estimation of the junction temperature of SiC MOSFETs based on the existing measured signals using their 3rd-quad characteristics triggered by the introduced dual-gate bias (DGB) of the driver on the power converter board. The datasheet (i.e., test calibration) covers various electrical characteristic changes caused by thermal effects and the responses of different TSEPs under various operating current and gate voltage conditions (i.e., dual-gate bias). In addition, using a datasheet will save the development cycle from calibrating TSEPs using dedicated equipment, while the target temperatures represented by TSEPs for the SiC MOSFET and the intrinsic diode are identical. Therefore, by controlling the closure degree of MOS channels through gate-driven sequential pulses, the corresponding combined TSEP can be obtained based on the response current to determine the junction temperature accordingly.

## 2. Temperature Characterization

The gate-to-source voltage $V_{\mathrm{GS}}$ and the junction temperature $T_{j}$ of a SiC MOSFET determine the static relationship function between drain–source current $I_{\mathrm{DS}}$ and drain–source voltage $V_{\mathrm{DS}}$. When $V_{\mathrm{DS}}>0$ V, the current flows from the drain to the source, and the device operates in the first quadrant (1st quad). Conversely, if $V_{\mathrm{DS}}<0$ V, the $I_{\mathrm{DS}}$ flow is reversed, defined as $I_{\mathrm{SD}}$, and then the device works in the third quadrant. This paper uses a commercial SiC MOSFET (C3M0075120D) manufactured by Cree as the application medium for the proposed method.

### 2.1. Conduction in First Quadrant

The ON-resistance $R_{1st}$ in the first quadrant consists of the drain resistance $R_{D}$, the source resistance $R_{S}$, the drift region resistance $R_{d}$, and the channel resistance $R_{ch}$, which is the intrinsic resistance of MOSFET strongly dependent on $V_{\mathrm{GS}}$, $T_{j}$, and certainly the operating point $V_{\mathrm{DS}}$. However, the constant ohmic contact between the metal and heavily doped region forms constant $R_{S}$ unrelated to $V_{\mathrm{GS}}$ in the source terminal.

(1) $$R_{1st}=R_{ch}\left(V_{\mathrm{GS}},T_{j},V_{\mathrm{DS}}\right)+R_{d}\left(V_{\mathrm{GS}},T_{j},V_{\mathrm{DS}}\right)+R_{S}\left(T_{j}\right)+R_{D}$$

In addition, $R_{D}$ in (2) is divided into three components: substrate resistance $R_{sub}$, resistance in the undepleted accumulation region $R_{acc}$, and junction field-effect transistor (JFET) region resistance $R_{\mathrm{JFET}}$. Similarly, the contact resistance $R_{sub}$ between the highly doped substrate and drain metal is not related to $V_{\mathrm{GS}}$, but to $T_{j}$. $R_{acc}$ depends on the thickness of the depletion region induced by $V_{\mathrm{DS}}$.

(2) $$R_{D}=R_{acc}\left(V_{\mathrm{GS}},T_{j},V_{\mathrm{DS}}\right)+R_{sub}\left(T_{j}\right)+R_{\mathrm{JFET}}\left(V_{\mathrm{GS}},T_{j},V_{\mathrm{DS}}\right)$$

**Figure 1:**
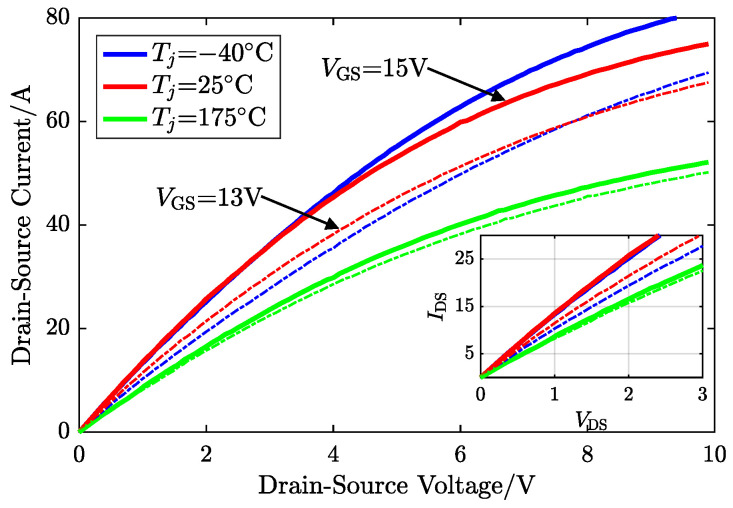
First-quadrant I-V curves of the SiC MOSFET affected by $V_{\mathrm{GS}}$ and $T_{j}$.

Therefore, the total resistance $R_{1st}$ depends on $V_{\mathrm{GS}}$, $V_{\mathrm{DS}}$ ( $I_{\mathrm{DS}}$ can be an alternative), and the estimated target $T_{j}$, as Figure 1 shows.

### 2.2. Conduction in Third Quadrant

**Figure 2:**
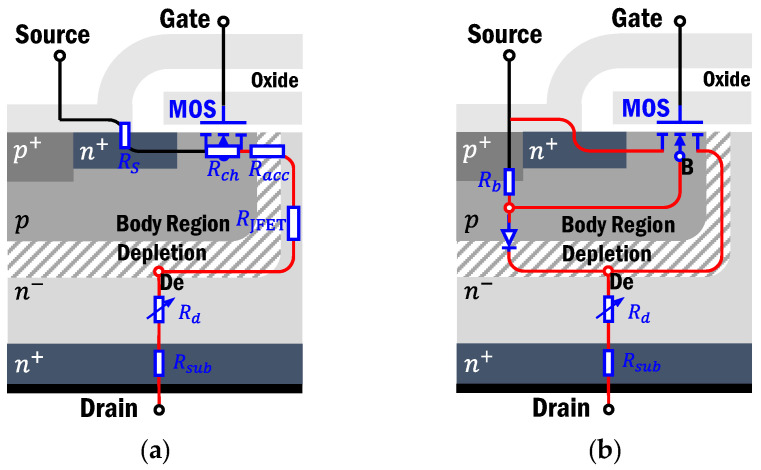
Equivalent circuit model in the different quadrants of the planar SiC MOSFET: (**a**) operating in the 1st quadrant; (**b**) operating in the 3rd quadrant.

Operating in the third quadrant, due to the structure of SiC MOSFETs, a p-i-n intrinsic diode is formed through p-base and n-drift, which are connected in an anti-parallel configuration across the conduction path of the MOSFET, known as the body diode, and $R_{b}$ is the p-base region resistance, as Figure 2 shows. For comparison, the equivalent model of a MOSFET operating in the first quadrant is also presented.

In addition, a parasitic n-p-n transistor would also be a constituent. However, the n+source and p-body are electrically shorted to suppress the formation of BJT. Therefore, the total source-to-drain voltage drop $V_{\mathrm{SD}}$ in the third quadrant is

(3) $$V_{\mathrm{SD}}=V_{S\text{-}\mathrm{De}}+I_{\mathrm{SD}}\cdot \left(R_{d}+R_{\mathrm{sub}}\right)$$

where De is the depletion region, located at the top of the n-drift and adjoined with the p-body. $V_{S\text{-}\mathrm{De}}$ consists of the voltage drops on $R_{b}$ and the voltage across the p-n junction, which may be equivalent to the parallel voltage drop on the inverse channel resistance. $I_{\mathrm{SD}}$ is the total source-to-drain current.

(4) $$I_{\mathrm{SD}}=I_{ch,3rd}+I_{bd}+I_{npn}$$

where $I_{bd}$ is the body diode current, $I_{ch,3rd}$ is the channel current in the third quadrant, and $I_{npn}$ is the parasitic npn current, which is inevitably activated in the third quadrant. However, due to the extremely low emitter injection efficiency, $I_{npn}$ is small and can be ignored. Therefore, two possible paths exist for the reverse current to flow. The first is the inverse MOS channel path, and the second is through the parallel body diode. The dominance between the two is primarily determined by $V_{\mathrm{GS}}$ and $V_{\mathrm{SD}}$.

**Figure 3:**
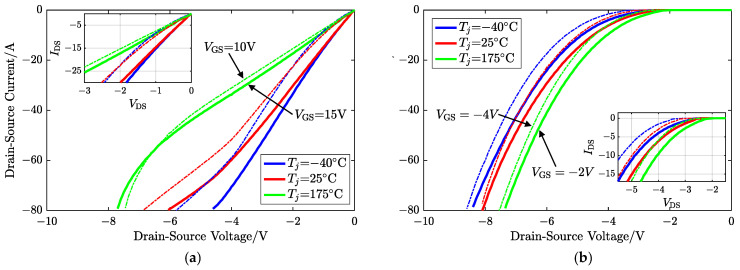
Comparison of I-V curves in the 3rd quadrant of the SiC MOSFET affected by different gate voltage biases: (**a**) positive $V_{\mathrm{GS}}$ and (**b**) negative $V_{\mathrm{GS}}$.

If positive, $V_{\mathrm{GS}}$ exceeds the 3rd-quad threshold voltage $V_{\mathrm{th}3}$, the thickness of the depletion layer surrounded by electron-rich regions becomes thicker, and an inversion layer in the channel region is formed. Normally, the body diode path has a lower resistance compared to the MOS channel, and hence, the body diode characteristics dominate in the high-current-and-high-voltage region in the third quadrant. In other words, if the voltage drop across the body diode is insufficient for forward bias, the channel characteristics dominate, as shown in Figure 3a. For negative $V_{\mathrm{GS}}$, the gate bias is the gate-to-De voltage $V_{G\text{-}\mathrm{De}}$. When $V_{\mathrm{SD}}$ increases under the given $V_{\mathrm{GS}}$, $V_{G\text{-}\mathrm{De}}$ goes up while $V_{\mathrm{th}3}$ declines, and $V_{G\text{-}\mathrm{De}}$ eventually exceeds $V_{\mathrm{th}3}$. Thus, the MOS channel forms, and its formation is easier than in the first quadrant. Specifically, $V_{\mathrm{SD}}$ is high enough to overcome the barrier and form the current in the base, i.e., the channel. The body diode dominates the 3rd-quad current. For a certain $I_{\mathrm{SD}}$, negative $V_{\mathrm{GS}}$ is small enough and $V_{G\text{-}\mathrm{De}}$ cannot form the channel, and the hole current through the pn body becomes the only flow, as Figure 3b shows.

However, the proportion of the current components is influenced by the gate bias, operating region, and junction temperature, and the turn-ON voltage of the body diode is also a related function. Calculating the current components of the two paths is complex and difficult. Therefore, the current can be regarded as a whole for simplification. So, the overall voltage drop of a MOSFET operating in the third quadrant depends on $V_{\mathrm{GS}}$, $I_{\mathrm{SD}}$, and $T_{j}$.

### 2.3. Operating Quadrant in the Drive System Circuit

The entire electric drive system of EVs mainly consists of three parts: a high-power battery pack, a three-phase full-bridge inverter, and the stator winding of the traction motor. The internal resistive and inductive loads of the three parts are connected to form a circuit loop by different switching combinations of six SiC MOSFETs of the inverter. Since the bus voltage is the essential system signal and available, the electrical characteristics of the battery in the circuit are ignored. When all three half-bridges are involved in control and current flows, there are two types of conduction modes based on whether the upper half-bridge works, namely, “one-half-bridge conduction” and “two-half-bridge conduction”. The lower MOSFET of the remaining non-conducting half-bridge remains on, allowing the current to pass through. “Three-half-bridge conduction” has no current output, so it is ignored.

**Figure 4:**
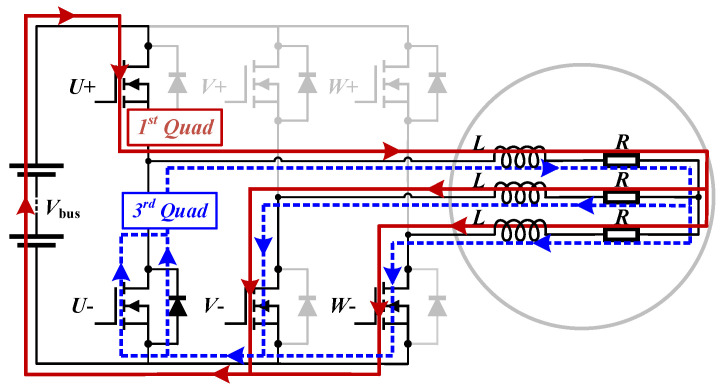
Circuit loops of “one-half-bridge conduction” in the drive system.

Due to the inductive component *L* in the stator winding and the 3rd-quad operating capability of MOSFETs, in the steady state, the system can be viewed as a buck converter with different switching combinations. The output voltage can be adjusted by the duty cycle of the upper half-bridge in the continuous condition. There are two current modes in one switching cycle corresponding to the operating quadrant of the MOSFET in the conducting half-bridge, where $I_{\mathrm{DS}}$ is defined as “forward” and then $I_{\mathrm{SD}}$ as “reverse”. Figure 4 shows the situation of “one-half-bridge conduction”.

Table 1 lists the MOSFETs, phase resistance, and phase inductance connected to the circuit in two conduction modes, as well as the operating quadrants of MOSFETs.

For different circuit loops, every half-bridge has a single MOSFET participating in it; thus, the total number is three. The same goes for the motor winding.

Compared to “one-half-bridge conduction”, in the mode of “two-half-bridge conduction”, two parallel lower half-bridge MOSFETs operate in the third quadrant and connect a forward-working MOSFET in series to accomplish freewheeling. In the forward current mode, the complementary MOSFETs of freewheeling are on. According to the series or parallel connection of half-bridges in the two conduction modes, two types of currents flow through power transistors. Starting from here, unless otherwise specified, the current subscript *s* represents the line current, and *p* stands for the phase current (and $i_{s}=2i_{p}$). In addition, the superscripts *f* and *r* stand for the “forward” and “reverse” current modes, respectively.

## 3. Triggering the 3rd-Quad Characteristics

In the practical application of traction inverters in EVs, the voltage level of the gate driver is designed based on the characteristics of the SiC MOSFET used. Generally, only two fixed values are employed: larger positive and negative gate biases. As mentioned in Section 2, a greater positive gate bias ($V_{\mathrm{GS}}>V_{th}$) can allow MOSFETs to conduct in the first quadrant and operate in the third quadrant as well for freewheeling, while the negative gate bias causes the MOSFET to be shut in the first quadrant. Due to the structural mechanism of the device, MOSFETs and intrinsic diodes share the same junction temperature, which determines their respective characteristics. Similarly, they are also gate-bias-dependent. The employed drive voltages in this paper are 15 V and −2 V for increasing universality, which means the gate-related behaviors have been determined, and are exhibited in Figure 1 and Figure 3a,b as solid lines. Hence, the proposed method utilizes the existing two levels of voltage on the driver side, defined as “dual voltage bias”, which controls the degree of MOS channel closure, to construct a combined TSEP for identifying the junction temperature.

### 3.1. MOS Channel Dominates

For safety reasons, the lower half-bridge of the inverter is generally triggered by a dead time to prevent a short-circuit, thus forming complementary driving pulses of the upper and lower half-bridges. Therefore, the freewheeling driving voltage of the lower half-bridge is the greater positive bias, where $V_{\mathrm{GS}}=15$ V. Thus, the channel behavior dominates.

For buck-converter-type circuits, under steady-state output, the change in inductance voltage in one switching cycle is approximately zero based on the volt-second balance. For the “forward” current mode of “one-half-bridge conduction”, $V_{L,s}$ on the transient line current $i_{L,s}^{f}$ can be expressed by

(5) $$\frac{3}{2}V_{L,s}=\frac{3}{2}L\frac{di_{L,s}^{f}}{dt}≃V_{\mathrm{bus}}-i_{L,s}R_{ds}-i_{L,p}R_{ds}^{*}-\frac{3}{2}i_{L,s}R$$

where *L* and *R* are the phase inductance and resistance of the stator winding, respectively. $i_{L,s}$ and $i_{L,p}$ stand for the steady-state current, where $i_{L,s}=2i_{L,p}$. $V_{\mathrm{bus}}$ is the bus voltage. $R_{ds}$ and $R_{ds}^{*}$ are the equivalent ON-state resistances in the first quadrant under respective operating currents.

In the “reverse” mode, the inductance attempts to maintain the current by reversing its polarity. $V_{L,s}$ can be expressed by $i_{L,s}^{r}$ (i.e., freewheeling current).

(6) $$\frac{3}{2}V_{L,s}=\frac{3}{2}L\frac{di_{L,s}^{r}}{dt}≃-i_{L,p}R_{ds}^{*}-i_{L,s}R_{sd}-\frac{3}{2}i_{L,s}R$$

where $R_{sd}$ is the equivalent ON-state resistance of the third quadrant under its current condition. Based on the volt-second balance, the total change in the continuous current in one switching cycle is approximately zero, i.e., $\Delta i_{L,s}^{f}=\Delta i_{L,s}^{r}$. The steady-state current is determined by the duty cycle *D* of the involved half-bridge, i.e., the proportion of the “forward” mode duration $\tau_{f}$ and the switching cycle $\tau_{\mathrm{SW}}$.

(7) $$\tau_{f}=D\cdot \tau_{\mathrm{SW}}=\tau_{\mathrm{SW}}-\tau_{r}$$

where $\tau_{r}$ is the period of the “reverse” mode. From (5)–(7), the steady-state current of the “one-half-bridge conduction mode” $i_{L,s,1hf}$ can be derived.

(8) $$i_{L,s,1hf}=\frac{DV_{\mathrm{bus}}}{DR_{ds}+0.5R_{ds}^{*}+\left(1-D\right)R_{sd}+1.5R}$$

In “two-half-bridge conduction”, although another parallel half-bridge participates in PWM control, the involved devices for the circuit in the “forward” current are identical to those in “one-half-bridge conduction”, as shown in Table 1. Hence, the inductance voltage expression is the same as (5). However, due to the different components through which current flows in the “reverse” mode, $V_{L,s}$ is given by

(9) $$\frac{3}{2}V_{L,s}=\frac{3}{2}L\frac{di_{L,s}^{r}}{dt}≃-i_{L,s}R_{ds}-i_{L,p}R_{sd}^{*}-\frac{3}{2}i_{L,s}R$$

where $R_{sd}^{*}$ is 3rd-quad ON-state resistance under $i_{L,p}$. While the MOS channel dominates, the steady-state output of the “two-half-bridge conduction” $i_{L,s,2hf}$ is

(10) $$i_{L,s,2hf}=\frac{DV_{\mathrm{bus}}}{R_{ds}+0.5DR_{ds}^{*}+0.5\left(1-D\right)R_{sd}+1.5R}$$

### 3.2. Body Diode Dominates

$V_{\mathrm{th}3}$ varies for different types of MOSFETs, $V_{\mathrm{SD}}$, and temperatures, but a negative gate bias is essential to prevent unintentional conduction. In this paper, to cover most situations of MOSFETs rather than only the diode current of the extreme case, the negative drive voltage provided by the system partially forms the MOS channel: viz., both paths have current. If the MOS channel needs to be completely closed (i.e., the inversion layer does not exist), a greater reverse bias may be required.

As the negative gate bias increases, the potential barrier grows with the depletion layer’s extension, increasing the pn junction’s turn-on voltage $V_{f}$. If $V_{\mathrm{SD}}>V_{f}$, the semi-open channel inversion layer is parallel to the body diode.

As shown in Table 1, the characteristics of the body diode need to be considered during the freewheeling phase. The phase inductance voltage is independent of the gate bias voltage in the “forward” current mode. Thus, the current change during the rising phase of the switching cycle is consistently derived from (5). In the “reverse” current mode, the line current change in the “one-half-bridge conduction” mode is

(11) $$\Delta i_{L,s,1hf}^{r}=\frac{V_{bd}+i_{L,s}R_{bd}+i_{L,p}R_{ds}^{*}+1.5i_{L,s}R}{1.5L}\cdot \tau_{r}$$

where $V_{bd}$ and $R_{bd}$ are the equivalent forward voltage and resistance of 3rd-quad characteristics, respectively. Similarly, for the “two-half-bridge conduction” mode, the current change with two parallel MOSFETs operating in the third quadrant is

(12) $$\Delta i_{L,s,2hf}^{r}=\frac{V_{bd}^{*}+i_{L,p}R_{bd}^{*}+i_{L,s}R_{ds}+1.5i_{L,s}R}{1.5L}\cdot \tau_{r}$$

Symbols with superscript asterisks correspond to the values under the phase current. The steady-state current can be derived for two conduction modes as follows:

(13) $$i_{L,s,1hf}=\frac{DV_{\mathrm{bus}}-\left(1-D\right)V_{bd}}{DR_{ds}+0.5R_{ds}^{*}+\left(1-D\right)R_{bd}+1.5R}$$

(14) $$i_{L,s,2hf}=\frac{DV_{\mathrm{bus}}-\left(1-D\right)V_{bd}^{*}}{R_{ds}+0.5DR_{ds}^{*}+0.5\left(1-D\right)R_{bd}^{*}+1.5R}$$

## 4. Combined TSEPs as a Temperature Inductor

### 4.1. TSEPs Based on the Datasheet

**Figure 5:**
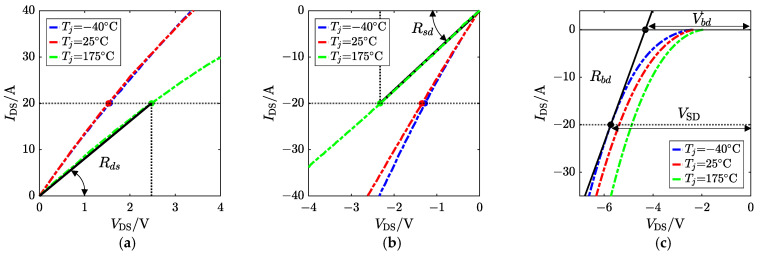
The definition of four TSEPs of a SiC MOSFET based on the figures in the datasheet: (**a**) $R_{ds}$, (**b**) $R_{sd}$, and (**c**) $V_{bd}$ and $R_{bd}$.

According to (8), (10), (13), and (14), the response current, as the inverter output, is determined by four electrical parameters of MOSFETs, which are related to the junction temperature, operating current, and gate bias. The gate bias, serving as the trigger prerequisite, has been fixed. Therefore, based on the datasheet, the TSEP functions $R_{ds}=f\left(i,T_{j}\right)$ related to the 1st-quad- and 3rd-quad-relevant $R_{sd}=g\left(i,T_{j}\right)$, $V_{bd}=h\left(i,T_{j}\right)$, and $R_{bd}=\phi \left(i,T_{j}\right)$ are defined. Their extraction method is shown in Figure 5.

$R_{ds}$ and $R_{sd}$ can be obtained by the ratio of $V_{\mathrm{DS}}$ and $I_{\mathrm{DS}}$, as shown in Figure 5a and Figure 5b, respectively. When the body diode dominates, the reverse voltage drop of the MOSFET $V_{\mathrm{SD}}$ is composed of $V_{bd}$ and the voltage drop of the equivalent ON-state resistance $R_{bd}$ in Figure 5c.

**Figure 6:**
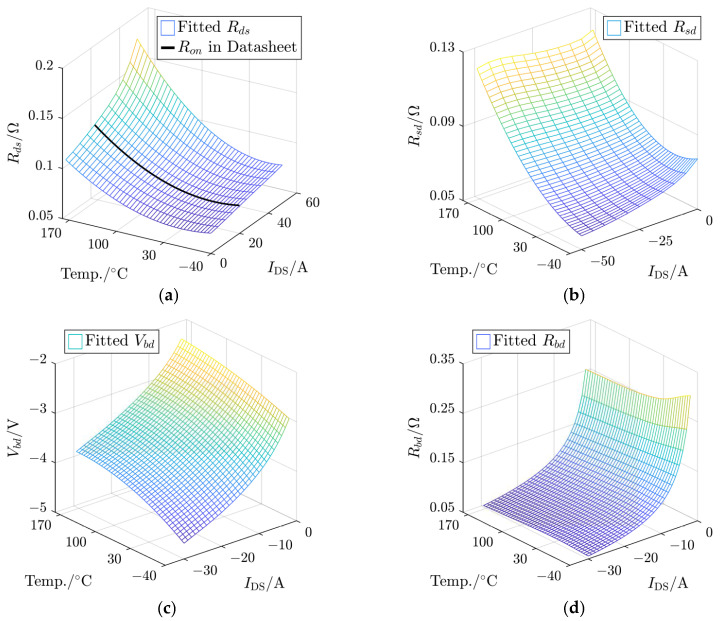
Polynomial-fitting surfaces for four TSEPs serving as the lookup table: (**a**) $R_{ds}$, (**b**) $R_{sd}$, (**c**) $V_{bd}$, and (**d**) $R_{bd}$.

Polynomial surface fitting can be carried out based on three temperature points, as Figure 6 shows.

Figure 6a shows that the ON-resistance $R_{\mathrm{on}}$ under $V_{\mathrm{GS}}=15$ V and $I_{\mathrm{DS}}=20$ A, provided by the datasheet, follows a reverse parabolic trend with temperature under the specified operating conditions. It demonstrates that the accuracy of the fitting results is satisfactory. In the third quadrant, the relationship between $R_{sd}$ and $R_{bd}$ with the operating current shows the opposite trend to the first quadrant. However, compared with $R_{bd}$, $R_{sd}$ has higher sensitivity to the junction temperature, as shown in Figure 6b,d. Figure 6c shows that $V_{bd}$ is highly sensitive to temperature and current changes.

As the measured current acts as a known input for four TSEP functions, the relationship curve between the TSEPs (resistance and voltage) and the junction temperature as the estimated target can be dynamically established under this output current. Based on the various steady-state currents derived in Section 3, the simultaneous equation to calculate the total resistance can be obtained by

(15) $$A\times {\left[\begin{array}{cccc} R_{ds} & R_{sd} & R_{bd} & R \end{array}\right]}^{T}=C$$

(16) $$\left[\begin{array}{cccc} \left(i_{L,s}\right)+\frac{1}{2}D\left(i_{L,p}\right) & \frac{1}{2}\left[1-D\right]\left(i_{L,p}\right) & 0 & \frac{3}{2}\\ D\left(i_{L,s}\right)+\frac{1}{2}\left(i_{L,p}\right) & \left[1-D\right]\left(i_{L,s}\right) & 0 & \frac{3}{2}\\ \left(i_{L,s}\right)+\frac{1}{2}D\left(i_{L,p}\right) & 0 & \frac{1}{2}\left[1-D\right]\left(i_{L,p}\right) & \frac{3}{2}\\ D\left(i_{L,s}\right)+\frac{1}{2}\left(i_{L,p}\right) & 0 & \left[1-D\right]\left(i_{L,s}\right) & \frac{3}{2} \end{array}\right]\left[\begin{array}{c} R_{ds}\\ R_{sd}\\ R_{bd}\\ R \end{array}\right]=\left[\begin{array}{c} DV_{\mathrm{bus}}/i_{L,s}\\ DV_{\mathrm{bus}}/i_{L,s}\\ DV_{\mathrm{bus}}/i_{L,s}-\left[1-D\right]V_{bd}\left(i_{L,p}\right)/i_{L,s}\\ DV_{\mathrm{bus}}/i_{L,s}-\left[1-D\right]V_{bd}\left(i_{L,s}\right)/i_{L,s} \end{array}\right]$$

where the first two equations describe the MOS channel dominating in different conduction modes, and the latter two represent the diode dominating. The matrix *A* stands for the resistance-related coefficients and the corresponding input value of the TSEP function, i.e., in brackets. In (16), the first and third rows express the “two-half-bridge conduction” mode, and the second and fourth rows represent “one-half-bridge conduction”, here omitting the subscript. In addition, if the gate bias or conduction mode is inconsistent, the current in different rows is diverse even at constant *D*. The matrix *C* is the output matrix, which calculates the equivalent resistance by system-supplied voltage and output current.

According to (8), (10), (13), and (14), the current is dependent on the duty cycle *D* as a control variable and TSEPs, which in turn rely on the current to form a mutual coupling. Therefore, the current is a variable input for TSEP functions, which can be decoupled by measuring the mean value of the steady-state response. In addition, all elements in the last column of the matrix *A* in (16) are the same: i.e., the resistance *R* coefficients of the stator winding are identical, and the phase resistance is not related to the current. Therefore, its effect can be eliminated by pairwise subtraction from the simultaneous equations. The phase resistance *R* independent of the current is ignored in the following.

### 4.2. Combined Resistance $R_{co}$ as TSEP of MOSFETs

The pairwise subtraction of (16) can be divided into three types: one is the sequential response of the two conduction modes under the dominance of MOS channels (the first minus the second row); the second only considers the dominance of the body diode triggered by the negative gate bias, i.e., subtracting the fourth row from the third; and finally, in the third type, both are involved, thus achieving DGB, that is, subtracting the first row from the third. To classify, the TSEP functions were placed on the same side of equations to form the equivalent resistance $R_{eq}$, not including *R*, and the mean value of the measured current was substituted into the three-dimensional TSEP functions for dimensionality reduction. The equation for estimating junction temperature can be summarized as follows:

(17) $${\left\{R_{eq,1}\right\}}_{\mathrm{cond}.\mathrm{mode}}^{\mathrm{gatebias}.\mathrm{dir}}-{\left\{R_{eq,2}\right\}}_{\mathrm{cond}.\mathrm{mode}}^{\mathrm{gatebias}.\mathrm{dir}}=R_{co}=D\left[\frac{V_{\mathrm{bus}}}{i_{s,1}}-\frac{V_{\mathrm{bus}}}{i_{s,2}}\right]$$

where the superscript of $R_{eq}$ represents the direction of the gate bias of the freewheeling MOSFET, and the subscript stands for the conduction mode, which are the trigger conditions for $R_{eq}$. The subscript number of $R_{eq}$ corresponds to the stage of the current pulse. Therefore, (17) introduces a two-stage current pulse trigger strategy with an identical *D*. The combined resistance $R_{co}$, as the proposed TSEP for a SiC MOSFET, is the difference between two $R_{eq}$.

The right side of (17) is defined as the “measurement side”, which is only related to the measured targets: $V_{\mathrm{bus}}$ and steady-state $i_{L,s}$. In the proposed method, both are existing and necessarily measured signals in the system. The left side of (17) is defined as the “table creation side”, which dynamically builds a lookup table based on the real-time current obtained.

(18) $$\begin{array}{ccc} \;R_{co}= & & \left[\begin{array}{c} {\left\{\;R_{ds}\left(i_{s}\right)\;+\;\frac{1}{2}DR_{ds}\left(i_{p}\right)\;+\;\frac{1}{2}\left[1\;\;-\;\;D\right]R_{sd}\left(i_{p}\right)\right\}}_{2hf}^{pos}\;\;-\;\;{\left\{DR_{ds}\left(i_{s}\right)\;+\;\frac{1}{2}R_{ds}\left(i_{p}\right)\;+\;\left[1\;\;-\;\;D\right]R_{sd}\left(i_{s}\right)\;\right\}}_{1hf}^{pos}\\ {\left\{\;R_{ds}\left(i_{s}\right)\;+\;\frac{1}{2}DR_{ds}\left(i_{p}\right)\;+\;\frac{1}{2}\left[1\;\;-\;\;D\right]R_{bd}\left(i_{p}\right)\;+\;\frac{\left[1\;-\;D\right]}{i_{s}}V_{bd}\left(i_{p}\right)\right\}}_{2hf}^{neg}\;\;-\;\;{\left\{DR_{ds}\left(i_{s}\right)\;+\;\frac{1}{2}R_{ds}\left(i_{p}\right)\;+\;\left[1\;\;-\;\;D\right]R_{bd}\left(i_{s}\right)\;+\;\frac{\left[1\;-\;D\right]}{i_{s}}V_{bd}\left(i_{s}\right)\;\right\}}_{1hf}^{neg}\\ {\left\{\;R_{ds}\left(i_{s}\right)\;+\;\frac{1}{2}DR_{ds}\left(i_{p}\right)\;+\;\frac{1}{2}\left[1\;\;-\;\;D\right]R_{sd}\left(i_{p}\right)\right\}}_{2hf}^{pos}\;\;-\;\;{\left\{R_{ds}\left(i_{s}\right)\;+\;\frac{1}{2}DR_{ds}\left(i_{p}\right)\;+\;\frac{1}{2}\left[1\;\;-\;\;D\right]R_{bd}\left(i_{p}\right)\;+\;\frac{\left[1\;-\;D\right]}{i_{s}}V_{bd}\left(i_{p}\right)\;\right\}}_{2hf}^{neg} \end{array}\right] \end{array}$$

where $i_{s}=i_{L,s}$ and $i_{p}=i_{L,p}$ for simplification. The formulas in the curly braces represent $R_{eq}$. Therefore, (18) provides theoretical formulas for three dynamic table-making approaches in the corresponding rows.

**Figure 7:**
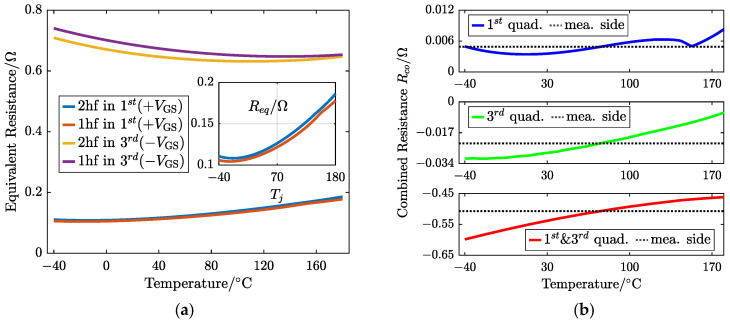
Dynamic creation of the two-dimension lookup table of resistance when the MOSFET is at $T_{j}$ = 75 °C with *D* = 0.03 based on the “spice” model: (**a**) $R_{eq}\left(T_{j}\right)$ with different conduction modes and gate biases; (**b**) three approaches to creating the $R_{co}$ table considering various combinations of gate bias directions.

Three dynamic lookup-table-making approaches were simulated at a specific set temperature ($T_{j}=75$ °C) with a small duty cycle *D* based on the spice model that the power SiC MOSFET supplier provided to verify the feasibility of the proposed junction temperature estimation method without additional measurement hardware and demonstrate the dynamic table-making process and its temperature estimation procedure, as shown in Figure 7.

The two-dimensional relationship curve between $R_{eq}$ and $T_{j}$ is obtained by introducing the mean value of the steady-state current into the TSEP functions under different triggering conditions, as shown in Figure 7a. The two adjacent curves (i.e., two $R_{eq}$) triggered by the positive gate bias are too close together, while the distance between the two triggered by the negative gate bias is relatively large. $R_{eq,2hf}$ and $R_{eq,1hf}$ under the negative gate bias are approximately four times higher than those under the positive, and their relationship with temperature tends to be opposite. Then, the final lookup table curve describing the relationship between $R_{co}$ and $T_{j}$ is formed by $R_{eq}$ pairwise subtraction (i.e., a two-stage current pulse injection), as shown in Figure 7b. The black dashed line represents the constant resistance value calculated by the “measurement side”, and it has an intersection point with the curve of the “table creation side”, whose x-axis value is the estimated value of the junction temperature. The top figure in Figure 7b shows that the approach only involved the positive bias; the curve has a small change of about 0.005 Ω in the entire temperature range and is not monotonic. Hence, there may be multiple solutions, and additional conditions must be introduced to determine the unique value, so this approach is excluded. However, the middle figure exhibits that the change range has expanded about 5 times compared to the positive bias, and both stages of this approach concern the negative gate bias in the third quadrant only. However, the order of the change magnitude shown in the figure below is $10^{-1}\;\Omega$, which is the largest among the three types of approaches.

**Figure 8:**
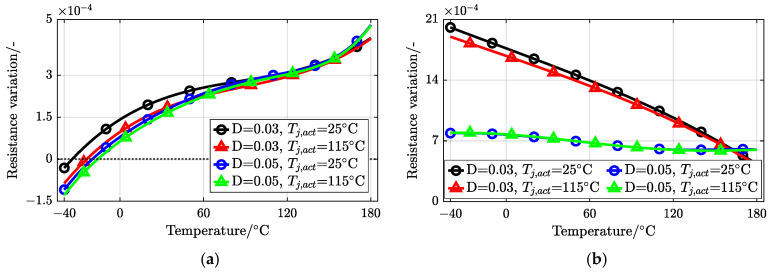
Derivatives of the dynamically created table $R_{co}$ on junction temperature $T_{j}$ under different test conditions (**a**) involving only the negative gate bias and (**b**) involving the dual-gate bias (pos. and nega.).

Figure 8 provides the monotonicity judgment of the latter two methods, comparing the derivative of $R_{co}$ on junction temperature under different set operating conditions (i.e., TSEPs at the set $T_{j,act}$) with two kinds of duty cycles *D*.

In Figure 8a, the method involving only the negative gate bias of the third quadrant has a derivative that crosses zero in the low-temperature range, so the established $R_{co}$ is not monotonic. Figure 8b shows that the derivative of $R_{co}$ involving two quadrants always remains positive. In addition, this approach has a greater variation with a smaller duty cycle. Therefore, this method is optimal among the three and satisfies application requirements.

## 5. Experimental Verification

The simulation fully demonstrates the feasibility and advantages of the proposed method. However, there are some differences between the “spice” model provided by the manufacturer and the actual situation, so the proposed algorithm was implemented and deployed into a self-built drive board to test the accuracy. This experimental rig is composed of an inverter board adopting six commercial-type discrete SiC MOSFETs and a Y-connected three-phase PMSM with a brake disc. The parameters are listed in Table 2.

**Figure 9:**
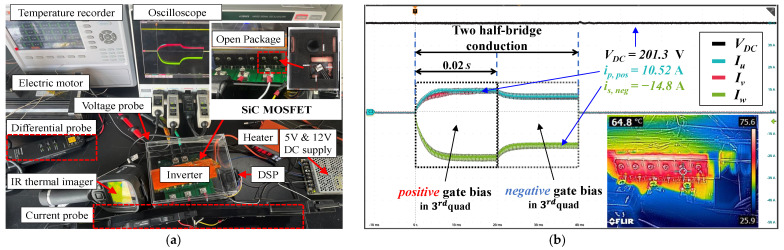
Test evaluations of the proposed method. (**a**) Experimental bench. (**b**) Measured signals of the oscilloscope and IR image for $T_{j}=64.8$ °C under D=0.05.

To ensure consistency in the inverter and accurately evaluate the algorithm, necessary static and dynamic tests were conducted on the MOSFETs before board-making to screen them. Therefore, it can be assumed that all output- and temperature-related characteristics of the six MOSFETs are consistent. According to (17), both the “table creation side” and the “measurement side” are highly correlated with the duty cycle, and the dead time set to prevent half-bridge short-circuits will reduce the actual conduction and freewheeling duration, which can cause estimation errors. Therefore, the actual duty-cycle calculation is

(19) $$D_{\mathrm{act}}=\left(1-\frac{\tau_{dt}}{D\cdot \tau_{sw}}\right)\cdot D$$

where $\tau_{dt}$ is the set dead time for half-bridges. Figure 9a shows the entire test bench used to evaluate and verify the proposed method for estimating the junction temperature of the MOSFET in the prototype drive system. Figure 9b provides the measurement results at $T_{j}=64.8$ °C, namely, the phase current, line current, and bus voltage under positive and negative biases.

The choice of −2 V as the negative driving voltage is not for a particular purpose, nor is it the recommended negative voltage level for the selected SiC power semiconductor. It is just a relatively random negative voltage because the used driving voltage for the SiC MOSFET may have an offset. By the proposed method, even utilizing the non-recommended voltage level can provide accurate estimation results. However, the negative driving voltage cannot be too small, as it introduces the influence of threshold voltage drift.

Firstly, several essential prerequisites need to be met before experimental validation. The electric brake is used to lock the rotor to eliminate the influence of mechanical transients on the current to simulate the start–stop or parking state of the vehicle. To better observe the actual junction temperature, the chip needs to be exposed, while only two SiC power MOSFETs of the *U* phase (i.e., on the identical half-bridge) are opened, considering that they retained their original packages in the comparison. The temperature of the wafer is measured using an infrared (IR) thermal imager and defined as the true value, as Figure 9b shows. Due to the two opened devices located on one half-bridge, the “two-half-bridge conduction” mode was adopted to compare the current with the unopened half-bridge to ensure that the measured junction temperature has a representation. When the current is the same, it indicates that the thermal states of the two half-bridges are consistent. Therefore, two half-bridges of the *U* and *V* phases are conducted simultaneously in two stages, whose distinction is the gate bias of the MOSFET in the third quadrant, resulting in apparent changes in current, as shown in Figure 9b.

In addition, the average value of the steady-state pulse current can prevent electrical transients and measurement errors. The time for a single-stage pulse (i.e., a conduction mode) is set to 0.02 s, and then the total time is 0.04 s. Therefore, the self-heating of the inverter is extremely small, the dual-gate bias strategy will not impact the temperature of the MOSFET, and the period to reach the steady state of the current is related to the motor phase inductance; hence, the time can even be further reduced. Similarly, the short-term current pulses do not heat the temperature-sensitive motor windings, so the resistances *R* of the two stages are identical, thus completely decoupling and eliminating their effects, as shown in (18).

**Figure 10:**
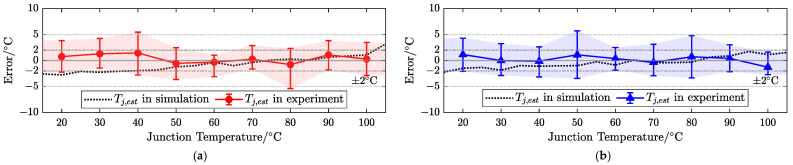
Error bars of temperature estimated using the proposed method under different conditions. (**a**) Duty cycle D=0.03. (**b**) Duty cycle D=0.05.

Using a heater and steady-state current injection, the MOSFETs of the inverter are uniformly heated to the specific measurement point of the junction temperature and stabilized, and the current junction temperature is estimated using the proposed dual-gate bias control strategy and identification algorithm. Based on the proposed algorithm, two-group control tests were carried out with different duty cycles (i.e., at different operating points), and the accuracy of junction temperature estimation was also evaluated through simulation and an experiment over a wide temperature range, as shown in Figure 10.

Each trial was performed five times under consistent temperature conditions to obtain its average. The temperature estimation method of the proposed dual-gate bias strategy has a small simulation error, which illustrates its feasibility. Moreover, on the test bench, it was implemented multiple times at various stable temperatures, and the maximum single errors were mostly within 5 °C and the mean error was below 2 °C when the duty cycle was 0.03 or 0.05.

Table 3 lists the root mean square error (RMSE) and mean absolute error (MAE) for dual-gate bias temperature estimation.

The results show that the proposed method has a lower dispersion degree and a mean error of about 2 °C. In Figure 8, under a wide temperature difference, the dynamically created TSEP-combined $R_{co}$ introduced by the proposed method has a similar trend and change quantity between −40 and 175 °C. Therefore, the sensitivity of $R_{co}$ based on a certain test point in the experiment can represent the temperature-sensitive characteristics of the device under a specific duty cycle. $R_{co}$ exhibits a high sensitivity positively correlated with temperature (roughly 5.67 $\times \;10^{-4}$Ω/°C for D=0.03 and 3.56 $\times \;10^{-4}$Ω/°C for D=0.05).

As shown in Table 3, the selection of the duty cycle has a relatively small impact on the accuracy of temperature estimation, which also indicates the broad adaptability of the proposed method and the low requirement for the current range. A larger duty cycle will inevitably introduce a higher steady-state current and heat, so a smaller duty cycle (i.e., a small current pulse) is superior and recommended.

**Figure 11:**
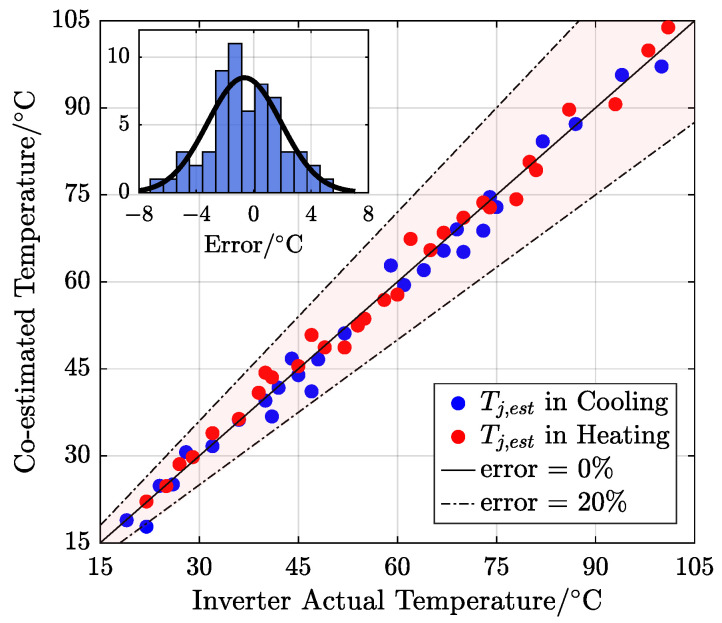
The deviation of the estimation for junction temperature $T_{j}$ under duty cycle D=0.03 in the heating/cooling process.

Therefore, to obtain a more comprehensive test at D=0.03, experimental verification was conducted during slow heating and cooling processes, and the results are in Figure 11.

The wide-range test from 15 to 105 °C showed that the estimation error of every measurement point is located within 15% relative error in centigrade. In addition, the histogram shows the error distribution, exhibiting a Gaussian distribution trend, and the maximum error is about 6 °C. These results fully demonstrate the accuracy of the proposed junction temperature estimation method for the SiC MOSFET triggered by the dual-gate bias of the third quadrant.

## 6. Advantages of the Proposed Method

Moreover, the advantage of the proposed method is verified by comparing it with the traditional method using current injection. The traditional method only considers the positive-bias freewheeling of the lower half-bridge MOSFET, so other conditions need to be used to supplement the lack of negative bias conditions, such as using two current pulses with different duty cycles or introducing two switching modes to construct the differences in current pulses.

**Figure 12:**
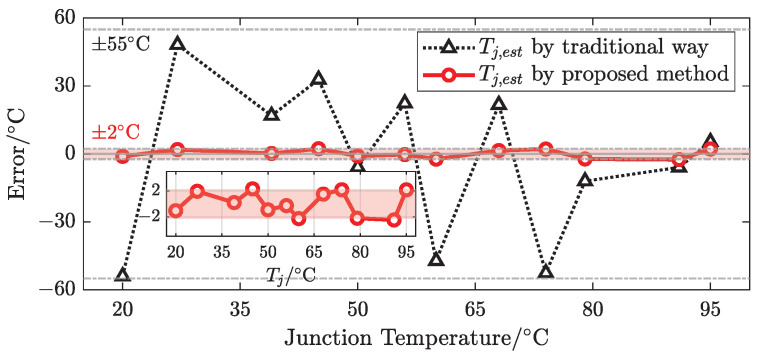
The comparison of estimation results between the proposed method and traditional method with D=0.03.

In order to simplify the complexity of the algorithm, the minimum duty cycle and a low current are used, but the nonlinearity of the device at low currents is also amplified. Furthermore, traditional methods ignore the correlation between the electrical characteristics of power semiconductors and operating points, to which TSEPs of the MOSFET are also sensitive. A comparison of the estimation errors between the proposed method and the traditional method was carried out based on the same test points, at which the experiment was repeated three times to obtain the mean value, as shown in Figure 12.

In Figure 12, the error of estimated $T_{j}$ compared with the traditional method fluctuates up to about 55 °C, while it can remain at about ±2 °C for the absolute error and below 7.2% for the relative error in centigrade with the proposed method. The comparison results indicate that the proposed method has good stability and high accuracy by introducing the dual-gate bias in the third quadrant and relevant electrical characteristics of the operating point, which is suitable for automobile applications.

Finally, a comprehensive comparison has been made between the proposed method in this article and previous TSEP works related to conduction characteristics, as shown in Table 4.

The methods in Table 4 all utilize TSEPs based on the conduction characteristics of power semiconductors for junction temperature estimation. The first two methods are for IGBTs, while the latter three are for SiC MOSFETs. Of the two methods for IGBTs, the method in [28] is for EV application, similar to this article, while that in [27] is suitable for washing machine drives, so the accuracy requirements may be different. All methods were calibrated or validated within roughly similar testing ranges. However, only a comprehensive calibration of TSEP was conducted in [20,21], lacking application verification on the bench. The combined TSEP proposed in this article has slightly lower sensitivity (which is also affected by different types of devices) compared to other methods but introduces nonlinear characteristics of the conduction current to establish $R_{co}$ dynamically, and it achieves high-precision junction temperature estimation without changing the existing circuit. The root mean square of the estimation error is the smallest, 2.28 °C.

## 7. Conclusions

This paper proposes an innovative technique for the sensorless estimation of the junction temperature of SiC MOSFETs in traction inverters by introducing the 3rd-quad characteristic using a dual-gate bias strategy and identifying the dynamically created TSEP-combined $R_{co}$. Innovatively considering the impact of the working current on the TSEPs of power semiconductors has improved estimation accuracy. The proposed method only requires the existing measured signals (i.e., $V_{\mathrm{bus}}$ and $i_{p}$), without any additional equipment, indicating that the proposed method has high portability and universality. The proposed method is independent of any temperature sensors. Due to its non-transient behavior, the winding effects being decoupled, and the introduction of the dual-gate bias strategy, it can be extended to various motor topologies and traction inverters composed of SiC MOSFETs. This article conducted comprehensive testing on a self-built test bench, and the experimental results showed that the proposed method has sufficient accuracy, with an average estimation error within 2 °C. In addition, compared with the traditional method, it demonstrates its superiority.

The proposed method is suitable for vehicle parking or start–stop states and can play a significant role in achieving the thermal safety of traction systems without changing hardware or affecting output power. It accurately provides the initial thermal state of the SiC MOSFET and can be used as input for other transient temperature rise estimation models. In addition, it can also serve as a redundant system for diagnosis to determine temperature sensor faults, thereby improving the reliability of EVs.

## Figures and Tables

**Table 1:** Components and the operating quadrant of the circuit through which the current flows in different conduction modes.

Conduction Mode	Current Mode	MOSFETs in 1st Quad		MOSFETs in 3rd Quad		RES. and IND. ^3^	
		S. ^2^	**P.** ^2^	S. ^2^	**P.** ^2^	S. ^2^	P. ^2^
One-half-bridge conduction	forward ^1^	1	2			1	2
	reverse ^1^		2	1		1	2
Two-half-bridge conduction	forward ^1^	1	2			1	2
	reverse ^1^	1			2	1	2

^1^ “Forward”: MOSFET operates in the 1st quadrant; “reverse”: MOSFET operates in the 3rd quadrant. ^2^ “S.”: in series; “P.”: in parallel. ^3^ “RES.”: phase resistance; “IND.”: phase inductance.

**Table 2:** Key parameters of the drive experimental rig.

**System**	**Parameter**	**Value**
SiC MOSFET Inverter	Type	C3M0075120D
	On-resistance, mΩ	75 ($V_{gs}$ = 15 V, $I_{ds}$ = 15 A)
	Gate–source voltage, V	+15/−2
	Carrier frequency, kHz	10
	Dead time, μ s	0.1
Traction Motor	Rated power, kW	3.8
	Rated current, A	17
	Pole pairs	4

**Table 3:** Estimation errors of junction temperature.

Validation Type	Duty Cycle *D*	Estimated $T_{j}$	
		RMSE/°C	MAE/°C
Simulation	0.03	1.98	1.67
	0.05	1.48	1.27
Experiment	0.03	2.28	1.83
	0.05	2.36	2.02

**Table 4:** Comparison of various methods for junction temperature estimation in [20,21,27,28].

Methods	TSEPs	Test Temp. Range/°C	Sensitivity * $10^{-3}$/°C	Intro. Current Nonlinearity	Additional Device	Est. Error *RMSE*/°C
Lu [28]	$R_{\mathrm{on}}$,$V_{\mathrm{drop}}$	10∼85	0.063Ω, −1.44V	no	no	3.28
Dianov [27]	Δ $U_{\mathrm{inv}}$	20∼120	≃0.79 V	no	no	≃4.09
Zhang [20]	$R_{\mathrm{ds},\mathrm{on}}$($V_{\mathrm{ds}}$),$V_{\mathrm{sd}}$	20∼110	0.81V, 1.915V	yes	yes	-
Kestler [21]	$V_{\mathrm{sd}}$	20∼120	−2.2V	yes	yes	-
This article	$R_{co}$	15∼105	0.57Ω	yes	no	2.28

* The unit of sensitivity is mV/°C or mΩ/°C.

## Data Availability

The data can be accessed from this manuscript.
